# Case report: morbid obesity and lithotomy positioning: a high-risk triad with GCS leading to severe rhabdomyolysis with impending acute compartment syndrome following ovarian cancer cytoreduction

**DOI:** 10.1186/s12893-026-03765-8

**Published:** 2026-04-23

**Authors:** Xiaoru Sun, Weimin Tao, Zhendong Xu

**Affiliations:** Department of Anesthesiology and Critical Care, Obstetrics and Gynecology Hospital, School of Medicine, Tongji University, Shanghai, 201204 China

**Keywords:** Venous Thromboembolism, Graduated compression stockings, Rhabdomyolysis, Compartment Syndromes, Morbid obesity

## Abstract

**Background:**

Venous thromboembolism (VTE) prophylaxis with graduated compression stockings (GCS) is a standard of care in gynecologic oncology surgery; however, the mechanical risks in specific patient populations require critical attention.

**Case presentation:**

A 24-year-old morbidly obese patient (BMI 41.7 kg/m²) with ovarian carcinoma developed severe rhabdomyolysis and impending acute compartment syndrome (ACS) after a 7.5-hour fertility-preserving cytoreduction performed in the Trendelenburg position with lithotomy, during which routine GCS were applied. Postoperative symptoms included severe bilateral leg pain, swelling, and myoglobinuria. Laboratory studies revealed extreme elevations in creatine kinase (peak CK 22,476 U/L), myoglobin (3,852 ng/mL), and lactate dehydrogenase, alongside hyperkalemia and hypocalcemia. A delayed initial CK reading due to hemolysis postponed targeted therapy. Aggressive conservative management, focusing on hydration and urine alkalinization, successfully resolved the rhabdomyolysis without the need for fasciotomy.

**Conclusions:**

While mechanical prophylaxis is vital, the convergence of morbid obesity, prolonged lithotomy positioning, and GCS application creates a high-risk triad that can precipitate severe rhabdomyolysis with impending ACS. Distinguishing severe rhabdomyolysis from established ACS is critical for guiding medical versus surgical management. Clinicians should maintain a low threshold for measuring intracompartmental pressures in symptomatic patients to prevent permanent neuromuscular damage or unnecessary surgical interventions.

## Background

Severe rhabdomyolysis and acute compartment syndrome (ACS) following prolonged gynecologic oncology surgery in the lithotomy position represent rare yet limb-threatening complications. Patients with gynecologic malignancies inherently face elevated venous thromboembolism (VTE) risks—exacerbated by both cancer-related hypercoagulability and intraoperative positioning—making combined pharmacomechanical prophylaxis the standard of care [1, 2]. Crucially, graduated compression stockings (GCS) remain a cornerstone of mechanical prevention due to their presumed safety and practical feasibility [3].

However, emerging evidence suggests that in specific high-risk scenarios—particularly morbid obesity coupled with extended lithotomy positioning—GCS may potentially contribute to a severe perfusion crisis by impairing venous return and promoting muscle ischemia [4]. Herein, we report a practice-changing case: a 24-year-old woman with a BMI of 41.7 kg/m² who developed severe rhabdomyolysis with impending ACS following a 7.5-hour fertility-sparing cytoreduction in the Trendelenburg position with lithotomy, during which routine GCS were applied. This case illuminates a previously underappreciated hazard: GCS-induced microcirculatory compromise in obese patients undergoing prolonged pelvic surgery, challenging the universal application of these garments without contextual risk assessment.

## Case presentation

### Patient profile

A 24-year-old female with morbid obesity (BMI 41.7 kg/m²) presented with suspected advanced ovarian cancer. Preoperative laboratory tests revealed an isolated D-dimer elevation (11.29 mg/L), indicating a hypercoagulable state.

### Surgical intervention

The patient underwent a fertility-sparing cytoreductive laparotomy lasting 7 h and 25 min. The prolonged duration was primarily due to extensive adhesiolysis necessitated by previous severe pelvic inflammatory disease. Intraoperatively, a frozen section of the right ovarian biopsy confirmed malignancy (Left adnexa, Malignant ovarian tumor, consistent with germ cell-derived neoplasm.), prompting full staging. The procedure included a left adnexectomy, right ovarian biopsy, multipoint peritoneal biopsy, omentectomy, para-aortic lymphadenectomy, and a Dixon procedure. No abnormal pelvic lymph nodes were found on exploration; therefore, pelvic lymphadenectomy was not performed. The Dixon procedure was performed due to direct tumor extension into the anterior rectal wall. The final histopathologic stage was reported as (Left adnexa) Ovarian yolk sac tumor with mature teratoma, involving (right ovarian biopsy), (omentum), and (pelvic peritoneum); no specific lesion identified in the fallopian tube. FIGO stage IIIc.

### Intraoperative factors

#### Position 

Trendelenburg position (15–30 degrees) with lithotomy. The calves were elevated 25 cm above heart level. This extended position was required to optimize pelvic exposure and facilitate both the abdominal cytoreduction and the trans-anal phases of the Dixon procedure simultaneously.

#### VTE prophylaxis 

Graduated compression stockings (Size XXL, fitted after measuring leg girth) were applied pre-anesthesia.

#### Hemodynamics 

Mean arterial pressure (MAP) was maintained at approximately 65 mmHg, with the exception of two transient hypotensive episodes lasting less than 10 min each.

Intraoperative pneumoperitoneum was not used; the procedure was performed via open laparotomy.

### Postoperative course and management

In the Intensive Care Unit (ICU), the patient developed severe bilateral lower limb pain, weakness, and significant swelling with erythema following GCS removal. Systemically, she presented with oliguria and tea-colored urine, indicative of myoglobinuria.

**Fig. 1:**
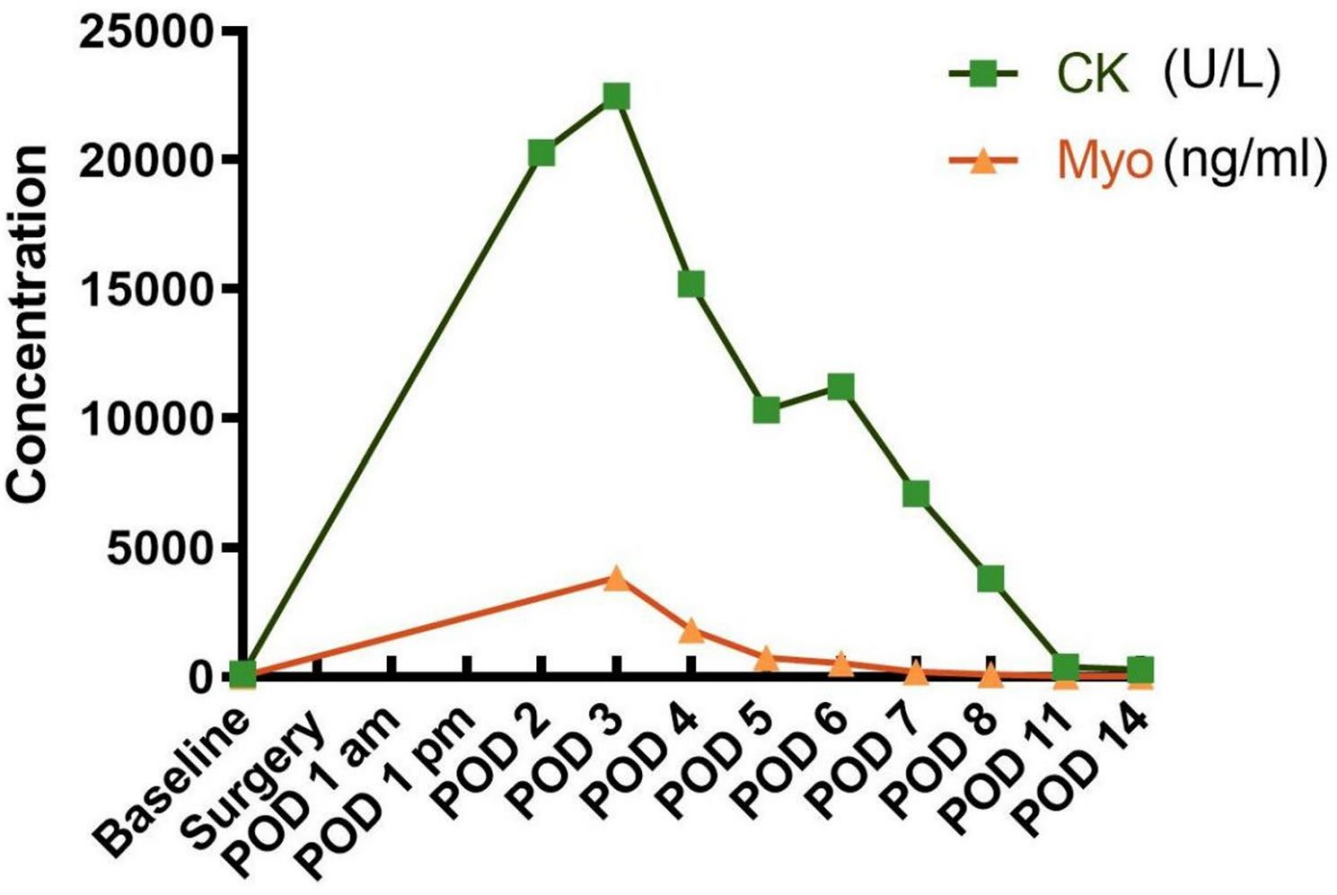
Changes in CK and MYO

**Fig. 2:**
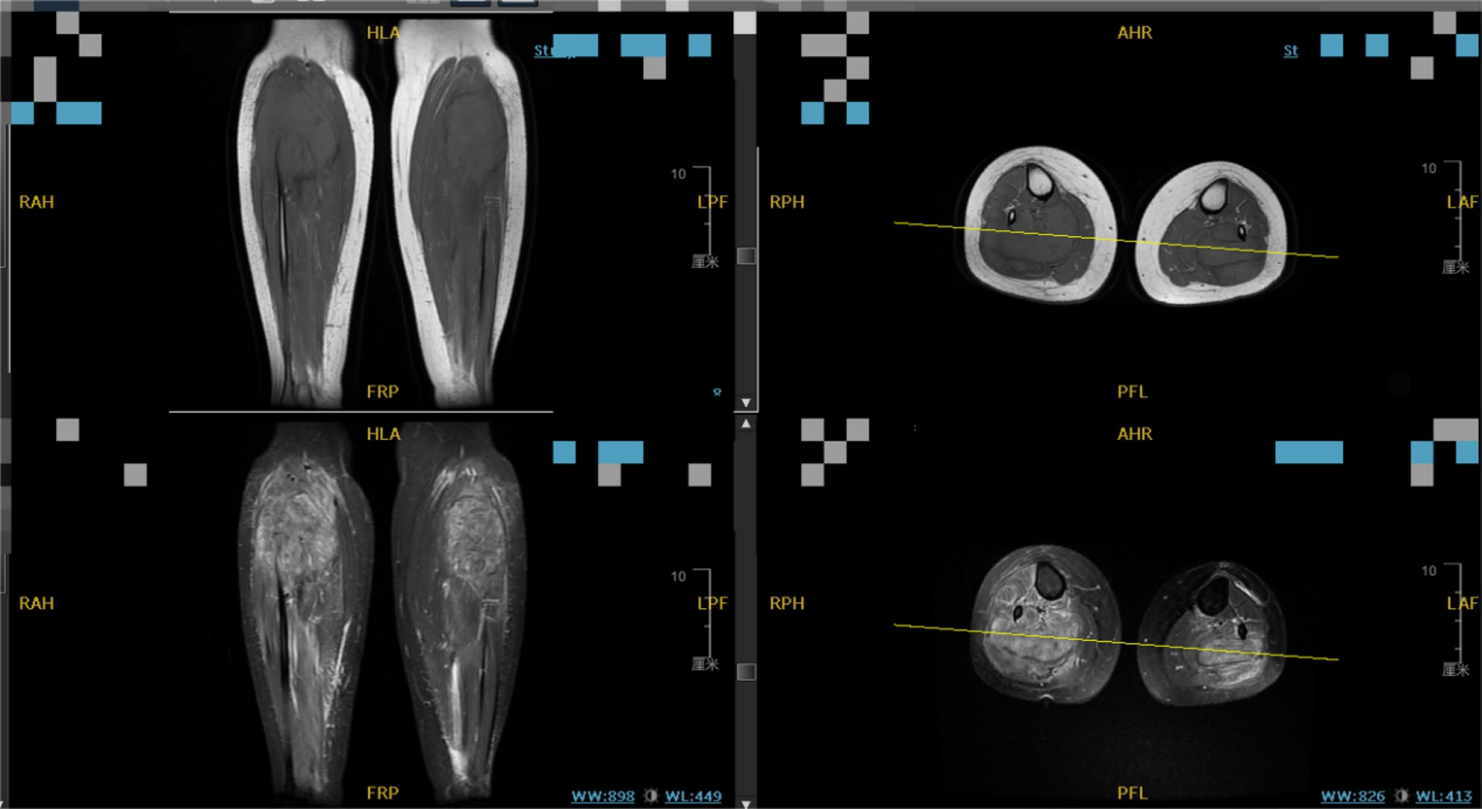
MRI imaging

An initial attempt to measure CK on postoperative day (POD) 1 was unsuccessful due to sample hemolysis. This diagnostic pitfall resulted in a 24-hour delay in obtaining the true value, which ultimately revealed extreme rhabdomyolysis: a CK level of 20,275 U/L (peaking later at 22,476 U/L) and myoglobin at 3,852.02 ng/mL (Table 1; Fig. 1). Additional laboratory abnormalities included hepatic injury (AST 597 U/L, ALT 148 U/L), renal stress (proteinuria 2+, hematuria 3+), hyperkalemia (5.4 mmol/L), and hypocalcemia (1.8 mmol/L). Magnetic Resonance Imaging (MRI) demonstrated diffuse subcutaneous edema and helped exclude important differential diagnoses including deep vein thrombosis, soft tissue infection, and primary myopathy, thus supporting the clinical diagnosis (Fig. 2).

**Table 1:** Laboratory testing of the patient

	CK (24-170U/L)	Myo (< 57.5 ng/ml)	LDH (135-247U/L)	K^+^ (3.5-5.3mmol/L)	Scr (53–115µmol/L)	AST (2–40 U/L)	Ca^2+^ (2-2.5 mmol/L)
Baseline	110	35.44	369	4.1	63	23	2.3
Day of surgery	-	-	1008	5.7	68	401	1.8
POD 1 06:00	-	-	964	5.4	78	597	1.8
POD 1 22:00	-	-	774	4.5	98	585	1.8
POD 2	20,275	-	690	4.0	93	546	1.8
POD 3	22,476	3852.02	868	4.1	84	674	1.9
POD 4	15,197	1818.25	988	4.0	75	577	2.0
POD 5	10,332	737.67	667	4.7	77	437	2.1
POD 6	11,229	529.03	750	4.4	73	457	2.1
POD 7	7089	187.98	734	4.3	77	369	2.1
POD 8	3805	87.92	677	4.2	84	257	2.2
POD 11	385	30.88	466	4.2	80	69	2.2
POD 14	278	29.25	393	3.9	77	30	2.3

Due to the absence of tense, woody compartments and the dominant biochemical markers of muscle breakdown without pulse deficits, a presumptive diagnosis of severe rhabdomyolysis with impending ACS was made. The therapeutic strategy aggressively targeted the prevention of rhabdomyolysis-induced acute kidney injury through continuous Ringer’s solution infusion, urine alkalinization (sodium bicarbonate), and diuresis promotion (furosemide). Serial monitoring of CK levels was conducted every 24 h. Following 14 days of aggressive active treatment, the patient’s biomarkers normalized, clinical symptoms significantly resolved, and permanent neuromuscular damage was prevented without the need for surgical fasciotomy.

## Discussion

While VTE prophylaxis is paramount in gynecologic oncology, this case exposes a critical paradox: mechanical compression intended for thromboprevention may potentially contribute to limb-threatening ischemic cascades in high-risk scenarios. Our patient developed severe rhabdomyolysis with impending ACS despite guideline-adherent GCS use. The convergence of multiple risk factors created a profound “perfusion crisis”.

First, the extended lithotomy position fundamentally reduces popliteal artery pressure [5]. While a Trendelenburg tilt might theoretically enhance venous return via hydrostatic mechanisms [6], in morbidly obese patients, the significant elevation in intra-abdominal pressure—exacerbated by abdominal packing and cephalad displacement of heavy visceral fat—creates a functional venous outflow obstruction that negates gravity-assisted venous return [7]. This venous stasis is further compounded by the lithotomy position, which inherently impairs arterial perfusion and venous drainage [8]. Elevated intra-abdominal pressure directly reduces peripheral perfusion pressure, thereby increasing the risk of muscle ischemia and rhabdomyolysis.

Second, the conical limb geometry characteristic of morbid obesity often causes standard thigh-high GCS to roll down or fold, creating a rigid band that acts as a tourniquet. This localized external compression easily exceeds capillary closure pressure in edematous tissue, critically impairing microcirculation and accelerating muscle necrosis, thereby explaining the extreme elevations in biochemical markers observed.

A major limitation of this report is that intracompartmental pressures were not directly measured. This lack of objective measurement reduces diagnostic certainty and prevents definitive distinction between severe rhabdomyolysis and early ACS. Clinical decisions were therefore based on clinical and biochemical findings rather than objective pressure data.

Crucially, it is vital to distinguish severe rhabdomyolysis from established, fulminant ACS. Standard surgical practice dictates that established ACS is a surgical emergency requiring immediate fasciotomy, as intracompartmental pressures exceeding capillary perfusion pressure lead to irreversible necrosis. Our patient’s successful conservative management implies she suffered from severe rhabdomyolysis with impending compartment syndrome, rather than definitive ACS. While aggressive hydration and renal protection are appropriate for rhabdomyolysis, our conservative success in this specific instance must not encourage delaying limb-saving surgery when definitive ACS is diagnosed.

### Clinical implications and preventive imperative

This case suggests that a paradigm shift in VTE prophylaxis for obese gynecologic oncology patients may be warranted. These findings highlight the importance of considering individualized risk assessment when BMI ≥ 35 kg/m² and prolonged lithotomy positioning (≥ 4 h) converge. We propose the following clinically relevant suggestions:

1. This case suggests that in high-risk patients, GCS may be avoided in favor of thigh-only intermittent pneumatic compression to prevent the tourniquet effect.
2. These findings highlight the value of limiting lithotomy duration to < 3 h with mandatory repositioning breaks (e.g., momentarily lowering the legs).
3. It is important to maintain diagnostic vigilance: clinicians should maintain a low threshold for measuring intracompartmental pressures in symptomatic patients, as distinguishing severe rhabdomyolysis from compartment syndrome is critical for guiding surgical versus medical management.

## Data Availability

All data generated or analyzed during this study are included in this published article.
